# Insights into the genome sequence of a free-living Kinetoplastid: *Bodo saltans *(Kinetoplastida: Euglenozoa)

**DOI:** 10.1186/1471-2164-9-594

**Published:** 2008-12-09

**Authors:** Andrew P Jackson, Michael A Quail, Matthew Berriman

**Affiliations:** 1 Wellcome Trust Sanger Institute, Wellcome Trust Genome Campus, Hinxton, Cambridgeshire, CB10 1SA, UK

## Abstract

**Background:**

*Bodo saltans *is a free-living kinetoplastid and among the closest relatives of the trypanosomatid parasites, which cause such human diseases as African sleeping sickness, leishmaniasis and Chagas disease. A *B. saltans *genome sequence will provide a free-living comparison with parasitic genomes necessary for comparative analyses of existing and future trypanosomatid genomic resources. Various coding regions were sequenced to provide a preliminary insight into the bodonid genome sequence, relative to trypanosomatid sequences.

**Results:**

0.4 Mbp of *B. saltans *genome was sequenced from 12 distinct regions and contained 178 coding sequences. As in trypanosomatids, introns were absent and %GC was elevated in coding regions, greatly assisting in gene finding. In the regions studied, roughly 60% of all genes had homologs in trypanosomatids, while 28% were *Bodo*-specific. Intergenic sequences were typically short, resulting in higher gene density than in trypanosomatids. Although synteny was typically conserved for those genes with trypanosomatid homologs, strict colinearity was rarely observed because gene order was regularly disrupted by *Bodo*-specific genes.

**Conclusion:**

The *B. saltans *genome contains both sequences homologous to trypanosomatids and sequences never seen before. Structural similarities suggest that its assembly should be solvable, and, although *de novo *assembly will be necessary, existing trypanosomatid projects will provide some guide to annotation. A complete genome sequence will provide an effective ancestral model for understanding the shared and derived features of known trypanosomatid genomes, but it will also identify those kinetoplastid genome features lost during the evolution of parasitism.

## Background

The Kinetoplastida (Euglenozoa) are unicellular flagellates that include the trypanosomatid parasites, most notably *Trypanosoma brucei*, *T. cruzi *and *Leishmania *spp. These organisms cause substantial mortality and morbidity in humans and their livestock worldwide as the causative agents of African sleeping sickness, Chagas disease and leishmaniasis respectively. *Bodo saltans *is a free-living heterotroph found worldwide in freshwater and marine habitats. It possesses the diagnostic kinetoplastid features, such as flagella sited within a specialised flagellar pocket, glycolytic processes confined to a dedicated organelle (the 'glycosome'), and the characteristic concentration of mitochondrial DNA at the base of the flagellum (the 'kinetoplast') [1,2]. When comparing trypanosomatid parasites with each other, or collectively with other eukaryotes, the value of *B. saltans *is as a non-parasitic near relative, (i.e., an 'outgroup'), that can illuminate their key evolutionary transitions. Five draft genome sequences exist for *Trypanosoma *spp. and four for *Leishmania *spp. [3-7]; these will be augmented with further strains and other non-human parasites in the coming years [8]. With such excellent comparative resources in place or in development, there is a critical need for a non-trypanosomatid outgroup. In effect, it will provide a model of the ancestral trypanosomatid to distinguish those derived parts of the parasite genomes (i.e., unique trypanosomatid adaptations) from those which are a legacy of the free-living ancestor. For instance, such a model will help to resolve whether trypanosomatids previously possessed an algal plastid from which 'plant-like' genes in trypanosomatid genomes are derived [9-11]. As a prelude to a complete *B. saltans *genome sequencing effort, this study sought to establish an initial understanding of the bodonid genome, its structure and content relative to the trypanosomatids.

The most recent kinetoplastid phylogeny has shown that trypanosomatid parasites are just one of many independent acquisitions of parasitism, indeed, a relatively minor component of total diversity [12-15]. Nonetheless, they are, naturally, the most important aspect of kinetoplastid diversity. Many features of their completed genome sequences emphasised the common ancestry of *T. brucei*, *T. cruzi *and *Leishmania *spp., especially with respect to gene repertoire and order [16], but their critical pathological differences were also evident at the genomic level. The three human parasites cause distinct diseases; their genomes contain enigmatic adaptations related to pathogenesis and immune evasion, for instance the bloodstream expression site in *T. brucei *from which its variant surface glycoproteins (VSG) are expressed [17,18], and surface antigen families in general [16]. Without an historical dimension, these features cannot be compared, nor understood in an evolutionary context. As it is among the closest bodonid relatives of the trypanosomatids [19], *Bodo saltans *is a suitable outgroup to address three principal comparative issues: i) understanding how human trypanosomatid parasites acquired their distinct pathological strategies; ii) understanding how the ancestral trypanosomatid became parasitic in terms of derived innovations (e.g., cell surfaces) and loss of genomic repertoire; iii) understanding how typical kinetoplastid features (e.g., glycosomes) evolved and how these might have been modified for parasitism.

Quite what to expect from a bodonid genome sequence is an open question. Beyond the basic kinetoplastid features named above, the biological differences between bodonids and trypanosomatids are striking. While *B. saltans *is a bacteriovore, especially prevalent in polluted waters or other environments with high bacterial densities [1], trypanosomatids are obligate parasites inhabiting a nutrient-rich, but ultimately hostile, host environment, and adept at exploiting their eutrophic environment to maximise proliferation and transmission. By contrast, *B. saltans *preys on bacterial cells [1,2] and is probably adapted for resource acquisition within its relatively oligotrophic environment. Although bodonids and trypanosomatids are all flagellates, trypanosomatids attach their single flagellum to the cell surface to generate motile force, whereas the anterior flagellum in *B. saltans *is modified with hair-like mastigonemes, which may assist prey location during feeding [2,20-22]. There are wider cytoskeletal differences also; the subpellicular microtubular cortex is instrumental in maintaining the numerous cell forms adopted by trypanosomatids [23], but is reduced in bodonids, (which lack complex developmental stages), to the region around the cytostome [2,24]. Perhaps most importantly for understanding the evolution of parasitism, we can expect substantial differences between trypanosomatid cell surfaces that function primarily to manipulate and frustrate the host immune response and bodonid membranes that are perhaps largely concerned with cellular homeostasis.

Rather than providing definitive answers to these questions, the preliminary sequence data presented here provides an initial insight into a few comprehensively resolved locations in the *B. saltans *genome, indicating what to expect from gene content and arrangement, and testing the feasibility of a complete sequence project. The sequence contigs were compared with corresponding regions in trypanosomatids (based on conserved gene order, where this existed), to examine gene content and the conservation of gene order (i.e., colinearity) and, therefore, the potential for using trypanosomatid genome sequences as scaffolds to assist assembly and annotation of the *B. saltans *sequence.

## Results

### Gene structure

**Figure 1:**
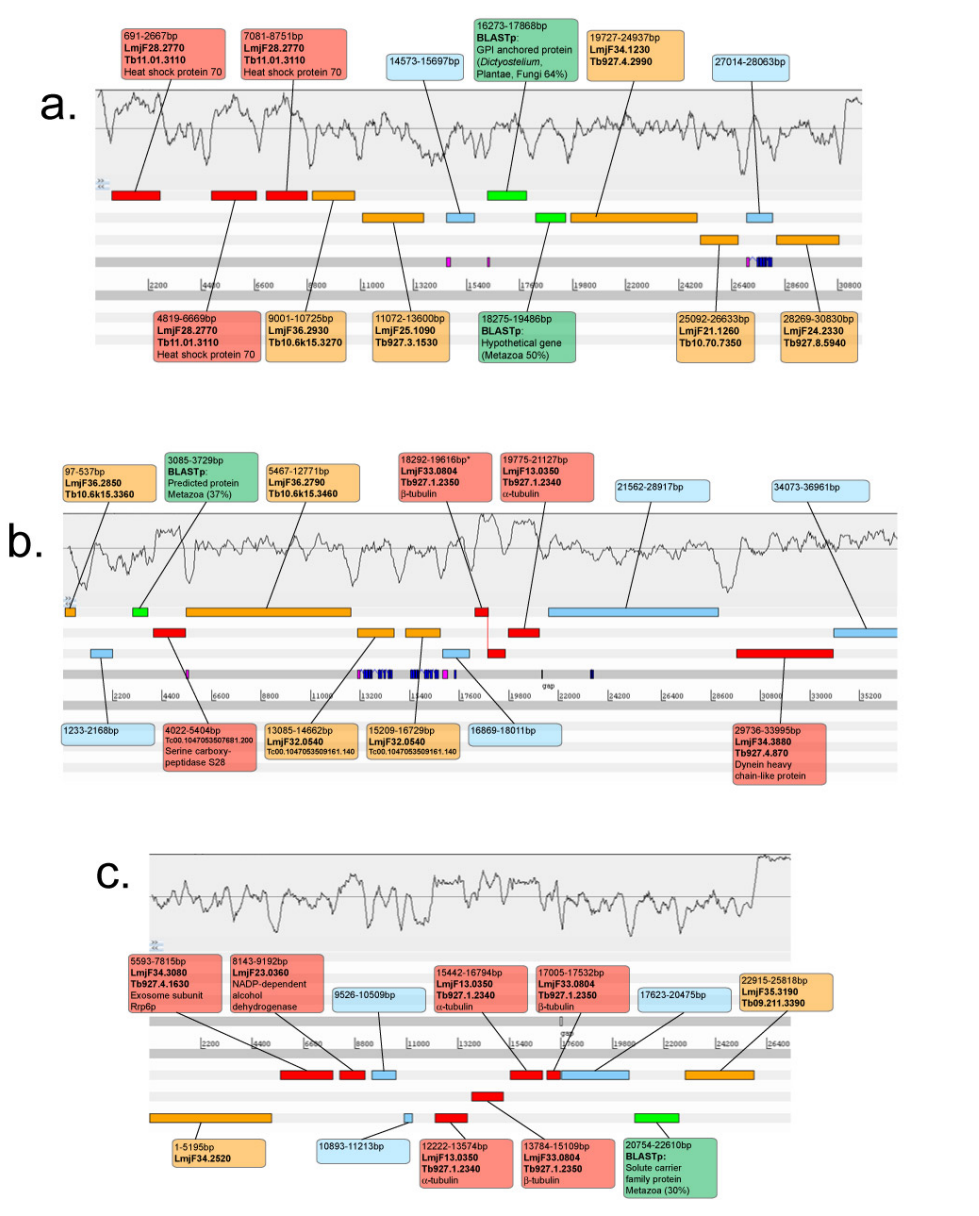
**Schematic representation of three regions of the *B. saltans *genome sequence, as shown in the Artemis genome browser**. Six reading frames are shown as parallel grey bars; scale in base-pairs. Base composition is plotted above. Putative coding sequences are shown as coloured boxes: red (homolog of trypanosomatid gene with known function), orange (homolog of hypothetical trypanosomatid gene), green (hypothetical gene with no trypanosomatid homolog but a positive functional match to a sequence database), blue (hypothetical gene with no matches to sequence databases). Labels attending these coding sequences contain the GeneDB identification numbers of homologous trypanosomatid genes where possible, or the description of homologous genes detected by BLAST comparisons (with % identity). Predicted transmembrane helices (blue) and signal peptides (purple) are shown on the DNA strands below the coding sequence. a. Clone '16k02' containing a tandem gene array of heat-shock protein 70. b. Clone '14l17' containing a tandem gene array of α- and β-tubulin. An asterisk * denotes a β-tubulin gene disrupted by a single base deletion at position 589. c. Clone '5m18' containing a second tandem gene array of α- and β-tubulin.

**Figure 2:**
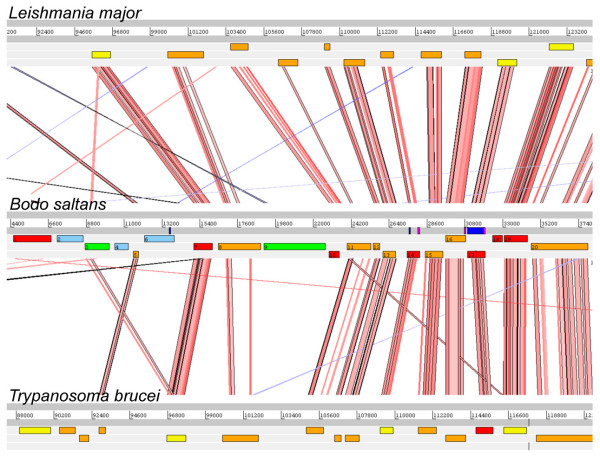
**Screenshot from the Artemis Comparison Tool (ACT), showing a 41.5 Kb fragment of *B. saltans *genome sequence (clone '45a12') and corresponding regions from chromosome 18 of *L. major *(top) and chromosome 6 of *T. brucei *(bottom)**. Key to *B. saltans *coding sequence annotation: 1. RNA-binding protein (homolog of Tb927.7.5380); 2. Hypothetical, no matches; 3. Serine-threonine protein kinase (Metazoa 46%, Plantae 43%); 4. Hypothetical, no matches; 5. Homolog of Tb10.61.3155; 6. Hypothetical lipase; 7. Serine-threonine protein kinase (Tb10.61.3140); 8. Homolog of Tb10.61.3130; 9. Possible ornithine decarboxylase (Bacteria 27%); 10. Dephospho-CoA kinase (Tc00.1047053511277.500); 11. Homolog of Tb10.61.3120; 12. Homolog of Tb10.61.3115; 13. Homolog of Tb10.61.3110; 14. DNAJ chaperone (Tb10.61.3100); 15. Homolog of Tb10.61.3080; 16. Homolog of Tb10.61.3070; 17. GPI-anchor transamidase (Tb10.61.3060); 18. Tubulin tyrosine ligase (Tb10.61.3050); 19. Ubiquitin-conjugating enzyme (Tb927.5.1000); 20. Homolog of Tb10.61.3040.

Clones were selected from the *B. saltans *fosmid library according to random end-sequences and positive results for specific PCR probes. Inserts from 12 fosmid clones were shotgun sequenced, comprising 0.403 Mbp in total and an average size of 33.6 Kbp. Table 1 describes the composition of the 12 contigs in terms of the affinity shown by each putative coding sequence to sequence databases. 178 putative coding sequences are specified; genes could be predicted by eye because of a definite elevation in GC content in coding regions. Subsequent matches to sequence databases showed these features to be correct. The boundaries between coding and flanking regions are marked by a transition from GC-rich to AT-rich signatures; the sequences shown in Figure 1 clearly demonstrate the GC troughs that appear between coding sequences. This pattern is repeated in other contigs, as shown in subsequent figures. Gene density is high relative to corresponding regions in the *L. major *and *T. brucei *genome sequences, reflected by the consistently short intercoding sequences across all contigs (average = 377.2 bp). Figure 2 compares the gene order of one region (average interceding sequence length = 439.7 bp) with positionally orthologous regions in *L. major *(average = 1480.6 bp) and *T. brucei *(average = 1129.4 bp); this, like most fosmid inserts, contains more genes in *Bodo *than in trypanosomatids.

**Table 1:** Description of genomic fragments sequenced in this study.

Clone	Probe^†^	Length (bp)	Genes by taxonomic affinity*					GenBank accession number
			K	E	P	U	TOTAL	
5 e 15	Rab1	35803	4	2	0	7	13	FJ168547
14 l 17	α-tubulin	38184	8	1	0	5	14	FJ168548
16 k 02	HSP70	32908	8	2	0	2	12	FJ168549
23 g 24	GPDH	37591	11	1	0	7	19	FJ168550
45 a 09		38203	7	1	1	5	14	FJ168551
45 a 12		41499	16	3	0	5	24	FJ168552
46 a 11		30067	8	3	1	3	15	FJ168553
5 m 18	β-tubulin	28046	9	1	0	3	13	FJ168554
93 d 02		18199	6	2	0	2	10	FJ168555
93 e 01		40818	12	2	0	3	17	FJ168556
96 f 03		38993	12	0	0	2	14	FJ168557
96 g 09		31028	5	2	0	6	13	FJ168558

**TOTAL**		**411339**	**106**	**20**	**2**	**50**	**178**	

Note: † Clones for which no probe is stated were selected after end-sequencing.* Genes were classified according to their affinity: kinetoplastid (K), other eukaryotic (E), prokaryotic (P) or unknown (U).

### Gene content

Table 1 shows that 106/178 coding sequences (59.6%) are homologs of known trypanosomatid genes. The percentage nucleotide identity between bodonid and trypanosomatid proteins varies greatly; genes of known conservatism display high identity (α-tubulin, 98%; β-tubulin, 99%; HSP70, 95%; GAPDH, 81%), but on average coding sequences are 44.38% identical and the most abundant identity class is 30–39%. Hence, most orthologs in these two classes have diverged by two-thirds or more. Of those coding sequences without trypanosomatid homologs, 20 show homology with other eukaryotes, 2 are of bacterial affinity, and the remainder (28.1%) are without matches to any database, i.e., *Bodo*-specific. Despite the bacterial contamination inevitable in DNA preparations (see methods), we can be certain that these bacterial-type coding sequences are not artefacts because they are present in fosmid inserts otherwise composed of eukaryotic sequences, and individual sequence clones span both the bacterial-type gene and surrounding eukaryotic-type sequence. Although present in *B. saltans*, some of the familiar genes intensively studied in trypanosomatids are found in novel contexts. Figure 1 describes tandem gene arrays of HSP70 and tubulin, which are found in locations unlike those in trypanosomatids. An alternating tandem array containing α and β-tubulin is found in two distinct inserts (Figure 1b and 1c); α and β-tubulin isoforms contain no amino acid differences but had dissimilar (unalignable) 3' untranscribed regions.

**Figure 3:**
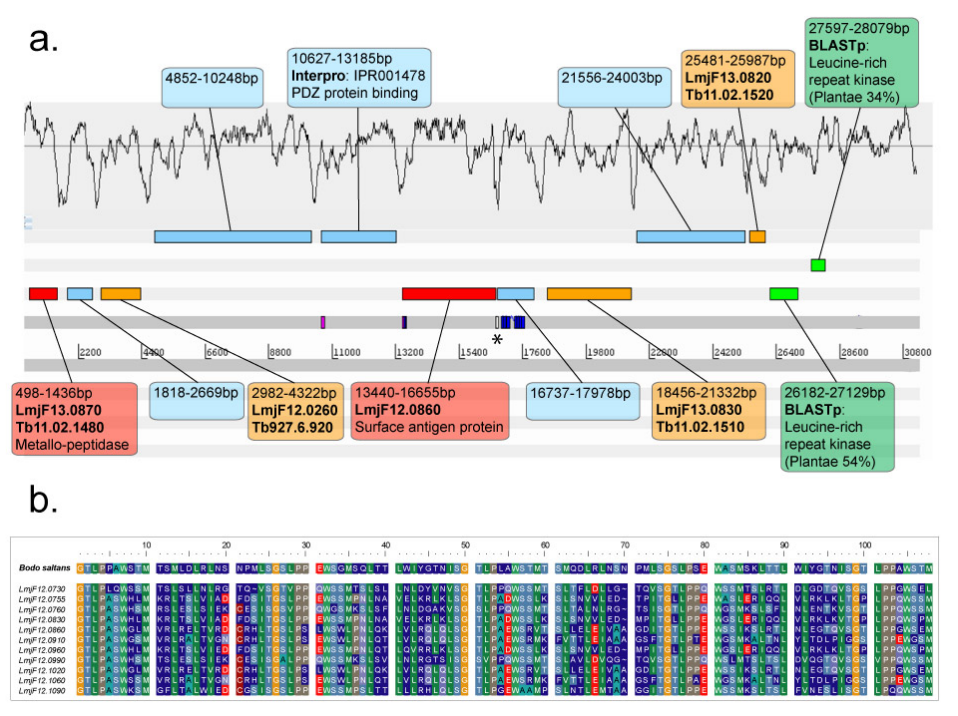
**a. Schematic representation of a 32.9 Kb fragment of *B. saltans *genome sequence (clone 96g09) in the Artemis genome browser**. The asterisk * denotes a physical sequence gap of uncertain length. b. Protein sequence alignment showing the repetitive region of 11 'surface antigen' proteins from *L. major *and their putative homolog from *B. saltans*, shown in panel a. (13440–16665 bp).

Coding sequences without trypanosomatid homologs were compared to sequence and structural databases (see Table 2). Many of the gene products are homologous to proteins beyond the Kinetoplastida, suggesting that they are core eukaryotic proteins subsequently lost from trypanosomatids; for example, contiguous genes in Figure 1a homologous to a GPI-anchored protein in plants and fungi (16273–17686 bp) and a hypothetical gene in Metazoa (18275–19486 bp). Gene products in other regions (not shown) contain protein domains known elsewhere, for example an ABC transporter protein (clone '5 e 15', 21065 bp) and a nucleotide-sugar transporter protein (clone '45 a 12', 41227 bp), strongly indicating that these are *Bodo*-specific members of ubiquitous gene families. Some otherwise uncharacterised hypothetical proteins are predicted to expressed on the cell surface. The region shown in Figure 3a is notable not only for the base composition of coding regions and admixture of trypanosomatid and *Bodo*-specific genes mentioned above, but also for a hypothetical protein (16737–17978 bp) with 7 predicted transmembrane helices and a signal peptide. Table 2 contains other examples of *Bodo*-specific hypothetical genes predicted to be surface expressed, including those shown in Figure 1a (27014–28063 bp) and b (16869–18011 bp).

**Table 2:** *Bodo*-specific hypothetical genes, with evidence of protein domains, transmembrane domains (TMH) signal peptides (SP) and affinities to sequence databases where available.

Clone	Position	Size (bp)	Interpro match	TMH	SP	Best BLASTp match	Best taxonomic match (% amino acid identity)
14 l 17	3085	645	Oxygenase (IPR005123)			Prolyl hydroxylase	Metazoa (53%)
	16869	1143	Zn-binding protein (IPR001841)	1			
	21613	7356	Leucine-rich repeat (IPR001611)	2			
16 k 02	14573	1125			Yes		
	16273	1596			Yes	GPI-anchored protein	Plantae/Fungi (64%)
	18275	1212				Hypothetical protein	Metazoa (50%)
	27014	1050		7	Yes		
23 g 24	391	1626	Pleckstrin-like (IPR001849)				
	7655	2781	Zinc metallopeptidase (IPR006025)				
	21361	23428				EF-hand protein	*Tetrahymena *(47%)
45 a 09	4161	535	Methyltransferase (IPR013216)				
	30026	1134	Phosphoribulokinase (IPR006083)				Bacteria (50%)
45 a 12	12191	1695	Lipase (IPR008265)	1			
	31394	455	Serine-Threonine protein kinase (IPR017442)			Protein kinase	Metazoa (46%)
	41227	264	Nucleotide-sugar transporter (IPR007271)	3	Yes		
46 a 11	211	753	Endonuclease (IPR001604)	1	Yes	Endonuclease	Bacteria (53%)
	1166	1416	Thioredoxin (IPR000866)			Nucleoredoxin	Plantae (53%)
	12286	1092				Fucosyltransferase	Plantae (36%)
	14365	765				Hypothetical protein	Metazoa (55%)
5 e 15	477	10617	Phosphatidylinositol-4-phosphate kinase (IPR003409)				
	14913	1296	Lyzosomal lipase (IPR006693)	1		Lipase	Metazoa (59%)
	16510	2199	Serine endopeptidase (IPR001254)			Serine protease	Plantae (46%)
	21065	546	ABC transporter (IPR013525)	4	Yes		
	32153	2613		1			
	35286	546	Zinc-finger protein (IPR001841)				
5 m 18	5577	1857	Sodium/hydrogen exchanger (IPR006153)	14	Yes	Sodium/hydrogen exchanger	Metazoa (50%)
	17069	321	Ankyrin repeat protein (IPR002110)				
93 d 02	5528	1758			Yes		
	7529	2589	Chloride channel (IPR0014743)	9		Chloride channel	Metazoa (56%)
	14924	1788	WD40 repeat (IPR001680)			Hypothetical protein	Plantae (56%)
93 e 01	56	5505		14			
	16439	1995	Forkhead associated protein (IPR000253)				
	18802	4260		8	Yes		
	24535	855	Protein tyrosine phosphatase (IPR000340)			Protein phosphatase	Plantae (56%)
96 g 09	10627	2559	PDZ protein binding motif (IPR001478)		Yes		
	16737	1242		7	Yes		
	26182	948	Leucine-rich repeat kinase (IPR001611)			Receptor-type protein kinase	Plantae (54%)
	27597	483	Leucine-rich repeat kinase (IPR001611)			Receptor-type protein kinase	Plantae (34%)

### Colinearity

The extent of conserved gene order, or colinearity, between bodonid and trypanosomatid genome sequences was assessed using the Artemis Comparison Tool (ACT, see methods). One region of excellent colinearity is shown in Figure 2 and, despite disruption by some eukaryotic genes not seen in trypanosomatids, this contig corresponds unmistakably with chromosome 18 in *L. major *and chromosome 10 in *T. brucei*. Conversely, the presence of so many non-trypanosomatid genes meant that colinearity disappears entirely in some locations, as shown in Figure 1. Across the 12 genomic regions however, both patterns were atypical; most regions shows brief patches of colinearity, perhaps 2 or 3 genes with conserved synteny, set among larger regions of *Bodo*-specific genes or homologs to trypanosomatid genes from elsewhere in the genome. In this sense, the sequence presented in Figure 3 is representative because several coding sequences are homologs of trypanosomatid genes on chromosomes 13 (*L. major*) and 11 (*T. brucei*); these are roughly colinear but the order is disrupted by genes present on other chromosomes or by *Bodo*-specific genes.

## Discussion

In this study, various locations in *B. saltans *genome, amounting to ~0.4 Mbp, were sequenced. Assuming that the bodonid genome is approximately the same size as a trypanosomatid haploid genome, i.e., 35–55 Mbp [16], these sequences comprise ~1% of the complete genome sequence, which will therefore contain roughly 14,000 genes. The success and utility of a *B. saltans *genome project will depend on its relationship with existing trypanosomatid genome sequences. This study shows that coding regions of the *B. saltans *genome share several structural features with trypanosomatids, indicating that the project is both feasible and likely to provide a useful comparative resource. Putative *B. saltans *genes lack introns, as in most trypanosomatid genes [3-5]. They display a conspicuous elevation in GC content, which will greatly assist gene finding. No evidence of strand-switching was observed in *B. saltans*, corroborating the view that it operates polycistronic transcription [25], i.e., transcription of many contiguous loci within a single nascent transcript [26-28], which is subsequently *trans*-spliced and polyadenylated to produce mature mRNA, as in trypanosomatids [29-33].

Although the arrangement of coding regions along the bodonid chromosome may be conserved with trypanosomatids, it is clear that gene order was not. The extent of conserved synteny, or rather colinear gene order, between bodonid and trypanosomatid genomes is of particular importance to the assembly of any *B. saltans *genome sequence. The coding regions presented here indicate that trypanosomatid genome sequences will be of limited value in the global assembly of a *B. saltans *genome sequence. Strict colinearity was not normally observed, if only because of the large number of *Bodo*-specific genes interposed between trypanosomatid homologs. Colinearity tended to persist over a distance of 3–5 genes, although some regions displayed conspicuous conservation (e.g., Figure 2), while others showed none at all (e.g., Figure 1). Therefore, this initial exploration of the *B. saltans *genome demonstrates that it should be possible to resolve a complete genome sequence, but, while the existing trypanosomatid resources will provide some useful guides for annotation, they could not be used as scaffolds for assembly, which should proceed *de novo*.

The purpose of a completed *B. saltans *genome sequence would be for understanding the evolution of trypanosomatid genome sequences. The mixture of familiar and novel features in the regions sequenced here indicates the value of a bodonid genome sequence in distinguishing trypanosomatid characters inherited from free-living ancestors (and still shared with them) from characters evolved since the origin of trypanosomatids. Hence, the first application would be in determining which parts of the trypanosomatid genome reflect the genomic legacy inherited from free-living ancestors, and show how they have been co-opted and modified for parasitism. Bodonid and trypanosomatid cells share various structural features, principally those that characterise kinetoplastid cells. Bodonids arrange their mitochondrial DNA in kinetoplasts, although their position within the cell differs from trypanosomatids [1], and conduct their glycolytic pathways within a dedicated organelle (the glycosome) [2]. Bodonids construct their flagella in a similar manner to trypanosomatids, but deploy them very differently [1]. While *B. saltans *uses one flagellum for movement and another for feeding, trypanosomatids flagella perform their motility function within the context of their sophisticated cell forms.

One might expect these structural similarities to be reflected at the genomic level. α- and β-tubulin, the proteins that facilitate the development of flagella in trypanosomatids, are known to be arranged in tandem gene arrays, with an alternating, heterotypic α-β array in *Trypanosoma *spp. and distinct, monotypic α and β arrays in *Leishmania *spp. [34-37]. Bodonids were shown to share the alternating conformation, suggesting that *Leishmania *spp. and their relatives had abolished the ancestral locus and evolved novel genomic repertoires [38]. However, two *B. saltans *regions containing tubulin in this study show that modification of tubulin repertoire has also occurred in *Trypanosoma*, since neither of the α-β arrays in *B. saltans *was found at the genomic position occupied in trypanosomes. This demonstrates the utility of the *B. saltans *genome in resolving the evolutionary causes of structural or compositional differences between trypanosomatid genomes.

The second application of a *B. saltans *genome sequence would be to identify which components of the free-living legacy have been lost from trypanosomatids, and therefore, how reductive genome evolution has contributed to the parasite genomes. Table 2 describes many predicted proteins identified in *B. saltans *that have no trypanosomatid homologs. Among these, mostly *Bodo*-specific, genes are membrane transporters, various protein kinases, and other proteins containing domains commonly associated with cell surfaces. These and other *Bodo*-specific proteins must include those metabolism pathways, intracellular transport, cellular signalling and subcellular structures that exist in free-living kinetoplastids, but which have been deleted during the evolution of parasitism. Many of these proteins will be widespread among eukaryotic lineages, as is evident in Table 2; yet we should also expect to encounter a considerable genetic repertoire unique to the Kinetoplastida and so entirely new.

Having identified those features of trypanosomatid genomes that reflect their free-living ancestry, a *B. saltans *genome sequence would also reveal the additions to each parasite genome; structures derived from existing genes and co-opted for novel uses, and genuinely novel genes involved in parasite-specific adaptations. These enigmatic genes include the numerous and diverse families of surface glycoprotein that form the protective coats around trypanosomatid parasites. *T. brucei*, *T. cruzi *and *L. major *each display highly derived and complex surface coats to frustrate host immunity, yet they differ in structure and substance and it is not known how each acquired its distinct solution to their common problem. Understanding the origins of these surface architectures will only be achieved with an historical perspective; one principal objective of a *B. saltans *genome project would be to identify the precursors of proteins such as VSG in *T. brucei*, mucins and trans-sialidase in *T. cruzi*, and proteophosphoglycans in *Leishmania *spp. (amongst others). A glimpse of this potential is seen in Figure 3, which includes a predicted protein with a complex 24 amino acid repeat (13440–16655 bp). The protein had a high affinity (42% amino acids identical) with a gene family on chromosome 12 in *Leishmania *spp., (currently annotated as 'surface antigens'), and a more distant affinity with proteophosphoglycans. Figure 3b shows a sequence alignment of the repeat domain from the *B. saltans *protein and its leishmanial homologs, where the level of amino acid identity rises to 50%.

## Conclusion

Thorough sequencing of a few locations in the *B. saltans *genome has revealed clear similarities with trypanosomatids, but has also shown that trypanosomatid genome sequences will not be effective guides for any complete bodonid project, due to significant differences in content and gene order. This mixture of familiar and novel features suggests that *B. saltans *will indeed provide an effective outgroup for comparisons of trypanosomatid parasites, and, as with the evolution of tubulin repertoire, the historical perspective to understand which aspects of trypanosomatid biology have been retained from their common ancestry, which have been lost, and what has been uniquely derived since.

## Methods

### Fosmid library preparation

A freshwater strain of *Bodo saltans *('Lake Konstanz'; courtesy of Dr Julius Lukes, University of South Bohemia, Czech Republic), was cultured in tap water in the presence of environmental bacteria. Bodonid cells were concentrated through a gentle centrifugation step (3,000 g for 2 minutes). Genomic DNA was prepared after resuspension of the pellet using phenol-chloroform extraction. This preparation contained a residuum of bacterial DNA. Genomic DNA was sheared and blunt-end repaired before being electrophoresed on a CHEF gel, from which the 25–40 kb band region was excised. The DNA was electroluted from the gel slice and ligated into a pCC1 fosmid vector (CopyControl Fosmid Kit; Epicentre Biotechnologies). Fosmid ligations were packaged into lambda bacteriophage (Gigapack XL2 Packaging Extract; Stratagene) and used to transform XL2-Blue MRF ultracompetent cells. Positive transformants were picked from chloramphenicol plates and cultured under drug selection. The *B. saltans *genome library contained 9600 individual clones (approximately 300 Mb).

### Clone selection and sequencing

As *B. saltans *cannot be grown axenically, 96 fosmid inserts were end-sequenced to examine the relative contributions of bodonid and bacterial DNA to the library. 16% of clones had end-sequences with affinity for eukaryotic coding sequences when compared to databases. Another 19% of clones had matches to bacterial sequences. Hence, although a larger proportion of end-sequences may have been genuine bodonid non-coding sequences (without representation in sequence databases), the library included a considerable, perhaps equal, component of bacterial DNA. Seven clones with positive end-sequence matches to trypanosomatid genes were sequenced in full (see below). Filters were prepared for the library by spotting bacterial culture on to a charged nylon membrane (Nytran Supercharge membrane: Schleicher and Schuell Bioscience) and lysing the cells; denaturation and fixation of the fosmid DNA produced a filter representing 8,832 genome fragments of 25–40 Kb. The filter was probed for five known *B. saltans *genes using radiolabelled PCR products, generated with the following primers: α-tubulin (F: AACGCSTGCTGGGAGYTGT; R: GTTGATRCCGCACTTGAAGCC; 1 kb), β-tubulin (F: AACCAGATCGGCTCNAAGTT; R: GATGTTGTTSGGGATCCACTC; 1 kb), Glyceraldehyde-6-phosphate dehydrogenase (F: CGGTCAAGGTAGGCATCAAC; R: TTGGGAAGGTTGTTCTGGAG; 800 bp), Heat shock protein 70 (F: TTCAAGAACGACCAGGTTGA; R: ACCAAGTCCGGCAACAATAG; 1.4 kb) and Rab1 (F: TTTGACAACCGBTACAAGGC; R: CCTTTGCGGACGTCTCAAAGTA; 500 bp). Clones corresponding to positive spots on the filter were cultured, and purified fosmid DNA was fully sequenced using a shotgun sequencing method to approximately 8× coverage.

### Assembly and analysis

Fosmid inserts were assembled using Phrap [39] and arranged within Gap4 [40]. PCR products were generated to close residual gaps between finished contigs. Finished sequence was annotated within Artemis [41,42] and coding sequences were initially defined by eye. Whole sequences were compared to EMBL sequence databases using both BLASTn and BLASTp algorithms. Coding sequences were scrutinised for possible transmembrane helices and signal peptides using TMHMM [43] and SignalP [44] respectively. Each coding sequence was checked for known protein domains using all options within the Interproscan suite [45]. Conserved synteny was assessed by aligning *B. saltans *contigs with *T. brucei *and *L. major *chromosomal regions using ACT [46] and existing trypanosomatid sequences downloaded from the GeneDB website [7].

## Abbreviations

ACT: Artemis comparison tool; PCR: Polymerase chain reaction; BLAST: Basic local alignment search tool; CDS: Coding sequence; IGS: Intergenic sequence; CHEF: Clamped homogeneous electric field.

## Authors' contributions

APJ prepared and probed the genomic library, assembled and annotated sequence contigs, analysed the data and prepared the manuscript. MAQ oversaw DNA preparation and sequencing. MB provided funds for sequencing and editorial guidance in producing the manuscript.

## References

[B1] Vickerman K, Preston TM, Lumsden WHR, Evans DA (1976). Comparative cell biology of the kinetoplastid flagellates. Biology of the Kinetoplastida.

[B2] Vickerman K, Patterson DJ, Larsen J (1991). Organization of the Bodonid Flagellates. The Biology of Free-living Heterotrophic Flagellates.

[B3] Berriman M, Ghedin E, Hertz-Fowler C, Blandin G, Renauld H, Bartholomeu DC, Lennard NJ, Caler E, Hamlin NE, Haas B, Bohme U, Hannick L, Aslett MA, Shallom J, Marcello L, Hou L, Wickstead B, Alsmark UC, Arrowsmith C, Atkin RJ, Barron AJ, Bringaud F, Brooks K, Carrington M, Cherevach I, Chillingworth TJ, Churcher C, Clark LN, Corton CH, Cronin A, Davies RM, Doggett J, Djikeng A, Feldblyum T, Field MC, Fraser A, Goodhead I, Hance Z, Harper D, Harris BR, Hauser H, Hostetler J, Ivens A, Jagels K, Johnson D, Johnson J, Jones K, Kerhornou AX, Koo H, Larke N, Landfear S, Larkin C, Leech V, Line A, Lord A, Macleod A, Mooney PJ, Moule S, Martin DM, Morgan GW, Mungall K, Norbertczak H, Ormond D, Pai G, Peacock CS, Peterson J, Quail MA, Rabbinowitsch E, Rajandream MA, Reitter C, Salzberg SL, Sanders M, Schobel S, Sharp S, Simmonds M, Simpson AJ, Tallon L, Turner CM, Tait A, Tivey AR, Van Aken S, Walker D, Wanless D, Wang S, White B, White O, Whitehead S, Woodward J, Wortman J, Adams MD, Embley TM, Gull K, Ullu E, Barry JD, Fairlamb AH, Opperdoes F, Barrell BG, Donelson JE, Hall N, Fraser CM, Melville SE, El-Sayed NM (2005). The genome of the African trypanosome *Trypanosoma brucei*. Science.

[B4] Ivens AC, Peacock CS, Worthey EA, Murphy L, Aggarwal G, Berriman M, Sisk E, Rajandream MA, Adlem E, Aert R, Anupama A, Apostolou Z, Attipoe P, Bason N, Bauser C, Beck A, Beverley SM, Bianchettin G, Borzym K, Bothe G, Bruschi CV, Collins M, Cadag E, Ciarloni L, Clayton C, Coulson RMR, Cronin A, Cruz AK, Davies RM, De Gaudenzi J, Dobson DE, Duesterhoeft A, Fazelina G, Fosker N, Frasch AC, Fraser A, Fuchs M, Gabel C, Goble A, Goffeau A, Harris D, Hertz-Fowler C, Hilbert H, Horn D, Huang YT, Klages S, Knights A, Kube M, Larke N, Litvin L, Lord A, Louie T, Marra M, Masuy D, Matthews K, Michaeli S, Mottram JC, Muller-Auer S, Munden H, Norbertczak H, Oliver K, O'Neil S, Pentony M, Pohl TM, Price C, Purnelle B, Quail MA, Rabbinowitsch E, Reinhardt R, Rieger M, Rinta J, Robben J, Robertson L, Ruiz JC, Rutter S, Saunders D, Schafer M, Schein J, Schwartz DC, Seeger K, Seyler A, Sharp S, Shin H, Sivam D, Squares R, Squares S, Tosato V, Vogt C, Volckaert G, Wambutt R, Warren T, Wedler H, Woodward J, Zhou SG, Zimmermann W, Smith DF, Blackwell JM, Stuart KD, Barrell B, Myler PJ (2005). The genome of the kinetoplastid parasite, *Leishmania major*. Science.

[B5] El-Sayed NM, Myler PJ, Bartholomeu DC, Nilsson D, Aggarwal G, Tran AN, Ghedin E, Worthey EA, Delcher AL, Blandin G, Westenberger SJ, Caler E, Cerqueira GC, Branche C, Haas B, Anupama A, Arner E, Aslund L, Attipoe P, Bontempi E, Bringaud F, Burton P, Cadag E, Campbell DA, Carrington M, Crabtree J, Darban H, da Silveira JF, de Jong P, Edwards K, Englund PT, Fazelina G, Feldblyum T, Ferella M, Frasch AC, Gull K, Horn D, Hou L, Huang Y, Kindlund E, Klingbeil M, Kluge S, Koo H, Lacerda D, Levin MJ, Lorenzi H, Louie T, Machado CR, McCulloch R, McKenna A, Mizuno Y, Mottram JC, Nelson S, Ochaya S, Osoegawa K, Pai G, Parsons M, Pentony M, Pettersson U, Pop M, Ramirez JL, Rinta J, Robertson L, Salzberg SL, Sanchez DO, Seyler A, Sharma R, Shetty J, Simpson AJ, Sisk E, Tammi MT, Tarleton R, Teixeira S, Van Aken S, Vogt C, Ward PN, Wickstead B, Wortman J, White O, Fraser CM, Stuart KD, Andersson B (2005). The genome sequence of *Trypanosoma cruzi*, etiologic agent of Chagas disease. Science.

[B6] Peacock CS, Seeger K, Harris D, Murphy L, Ruiz JC, Quail MA, Peters N, Adlem E, Tivey A, Aslett M, Kerhornou A, Ivens A, Fraser A, Rajandream MA, Carver T, Norbertczak H, Chillingworth T, Hance Z, Jagels K, Moule S, Ormond D, Rutter S, Squares R, Whitehead S, Rabbinowitsch E, Arrowsmith C, White B, Thurston S, Bringaud F, Baldauf SL, Faulconbridge A, Jeffares D, Depledge DP, Oyola SO, Hilley JD, Brito LO, Tosi LR, Barrell B, Cruz AK, Mottram JC, Smith DF, Berriman M (2007). Comparative genomic analysis of three *Leishmania *species that cause diverse human disease. Nat Genet.

[B7] Wellcome Trust Sanger Institute, Pathogen Sequencing Unit 'GeneDB' Interface. http://www.genedb.org.

[B8] Buck G, Berriman M, Donelson J, El-Sayed N, Kissinger J, Simpson L, Tait A, Teixeira M, Beverley S (2007). Pathogenomics of Trypanosomatid Parasites (White Paper). http://www.genome.gov/26525388.

[B9] Hannaert V, Saavedra E, Duffieux F, Szikora JP, Rigden DJ, Michels PA, Opperdoes FR (2003). Plant-like traits associated with metabolism of *Trypanosoma *parasites. Proc Natl Acad Sci USA.

[B10] Bodył A, Mackiewicz P (2008). Were class C iron-containing superoxide dismutases of trypanosomatid parasites initially imported into a complex plastid? A hypothesis based on analyses of their N-terminal targeting signals. Parasitology.

[B11] Leander BS (2004). Did trypanosomatid parasites have photosynthetic ancestors?. Trends Microbiol.

[B12] Dolezel D, Jirků M, Maslov DA, Lukes J (2000). Phylogeny of the bodonid flagellates (Kinetoplastida) based on small-subunit rRNA gene sequences. Int J Syst Evol Microbiol.

[B13] Simpson AG, Lukes J, Roger AJ (2002). The evolutionary history of kinetoplastids and their kinetoplasts. Mol Biol Evol.

[B14] Simpson AG, Gill EE, Callahan HA, Litaker RW, Roger AJ (2004). Early evolution within kinetoplastids (Euglenozoa), and the late emergence of trypanosomatids. Protist.

[B15] Simpson AG, Stevens JR, Lukes J (2006). The evolution and diversity of kinetoplastid flagellates. Trends Parasitol.

[B16] El-Sayed NM, Myler PJ, Blandin G, Berriman M, Crabtree J, Aggarwal G, Caler E, Renauld H, Worthey EA, Hertz-Fowler C, Ghedin E, Peacock C, Bartholomeu DC, Haas BJ, Tran AN, Wortman JR, Alsmark UC, Angiuoli S, Anupama A, Badger J, Bringaud F, Cadag E, Carlton JM, Cerqueira GC, Creasy T, Delcher AL, Djikeng A, Embley TM, Hauser C, Ivens AC, Kummerfeld SK, Pereira-Leal JB, Nilsson D, Peterson J, Salzberg SL, Shallom J, Silva JC, Sundaram J, Westenberger S, White O, Melville SE, Donelson JE, Andersson B, Stuart KD, Hall N (2005). Comparative genomics of trypanosomatid parasitic protozoa. Science.

[B17] Pays E (2006). The variant surface glycoprotein as a tool for adaptation in African trypanosomes. Microbes Infect.

[B18] Taylor JE, Rudenko G (2006). Switching trypanosome coats: what's in the wardrobe?. Trends Genet.

[B19] Heyden S von der, Cavalier-Smith T (2005). Culturing and environmental DNA sequencing uncover hidden kinetoplastid biodiversity and a major marine clade within ancestrally freshwater *Neobodo designis*. Int J Syst Evol Microbiol.

[B20] Karpov SA, Zhukov BF (1983). Ultrathin Structure of *Pleuromonas jaculans *Perty (Kinetoplastida, Zoomastigophorea). Prosteishie aktivnogo ila (Protozoa of the Active Silt).

[B21] Mitchell GC, Baker JH, Sleigh MA (1988). Feeding of a freshwater flagellate, *Bodo saltans*, on diverse bacteria. J Protozool.

[B22] Jezbera J, Horòák K, Šimek K (2005). Food selection by bacterivorous protists: insight from the analysis of the food vacuole content by means of fluorescence in situ hybridization. FEMS Microbiol Ecol.

[B23] Gull K (1999). The cytoskeleton of Trypanosomatid Parasites. Annu Rev Microbiol.

[B24] Brugerolle G, Lom J, Nohynkova E, Joyon L (1979). Comparison et evolution des structures cellulaire chez plusiers espèces de Bodonidés et Crytobiidés appartenant aux genres *Bodo*, *Cryptobia *et *Trypanoplasma *(Kinetoplastida, Mastigophora). Protistologica.

[B25] Santana DM, Lukes J, Sturm NR, Campbell DA (2001). Two sequence classes of kinetoplastid 5S ribosomal RNA gene revealed among bodonid spliced leader RNA gene arrays. FEMS Microbiol Lett.

[B26] Imboden MA, Laird PW, Affolter M, Seebeck T (1987). Transcription of the intergenic regions of the tubulin gene cluster of *Trypanosoma brucei*: evidence for a polycistronic transcription unit in a eukaryote. Nucleic Acids Res.

[B27] Flinn HM, Smith DF (1992). Genomic organisation and expression of a differentially-regulated gene family from *Leishmania major*. Nucleic Acids Res.

[B28] Wong S, Morales TH, Neigel JE, Campbell DA (1993). Genomic and transcriptional linkage of the genes for calmodulin, EF-hand 5-protein, and ubiquitin extension protein-52 in *Trypanosoma brucei*. Mol Cell Biol.

[B29] Van Hamme L, Pays E (1995). Control of gene expression in trypanosomes. Microbiol Rev.

[B30] Stiles JK, Hicock PI, Shah PH, Meade JC (1999). Genomic organization, transcription, splicing and gene regulation in *Leishmania*. Ann Trop Med Parasitol.

[B31] Campbell DA, Thomas S, Sturm NR (2003). Transcription in kinetoplastid protozoa: why be normal?. Microbes Infect.

[B32] Myler PJ, Beverley SM, Cruz AK, Dobson DE, Ivens AC, McDonagh PD, Madhubala R, Martinez-Calvillo S, Ruiz JC, Saxena A, Sisk E, Sunkin SM, Worthey E, Yan S, Stuart KD (2001). The *Leishmania *genome project: new insights into gene organization and function. Med Microbiol Immunol.

[B33] Monnerat S, Martinez-Calvillo S, Worthey E, Myler PJ, Stuart KD, Fasel N (2004). Genomic organization and gene expression in a chromosomal region of *Leishmania major*. Mol Biochem Parasitol.

[B34] Thomashow LS, Milhausen M, Rutter WJ, Agabian N (1983). Tubulin genes are tandemly linked and clustered in the genome of *Trypanosoma brucei*. Cell.

[B35] Seebeck T, Whittaker PA, Imboden MA, Hardman N, Braun R (1983). Tubulin genes of *Trypanosoma brucei*: A tightly clustered family of alternating genes. Proc Natl Acad Sci USA.

[B36] Maingon R, Gerke R, Rodriguez M, Urbina J, Hoenicka J, Negri S, Aguirre T, Nehlin J, Knapp T, Crampton J (1988). The tubulin genes of *Trypanosoma cruzi*. Eur J Biochem.

[B37] Das S, Adhya S (1990). Organization and chromosomal localization of beta-tubulin genes in *Leishmania donovani*. J Biosci.

[B38] Jackson AP, Vaughan S, Gull K (2006). Evolution of tubulin gene arrays in Trypanosomatid parasites: genomic restructuring in *Leishmania*. BMC Genomics.

[B39] Phrap sequence assembly program. http://www.phrap.org/phredphrapconsed.html.

[B40] Gap4: genome assembly program. http://staden.sourceforge.net/manual/gap4_unix_2.html.

[B41] Rutherford K, Parkhill J, Crook J, Horsnell T, Rice P, Rajandream MA, Barrell B (2000). Artemis: sequence visualization and annotation. Bioinformatics.

[B42] Berriman M, Rutherford K (2003). Viewing and annotating sequence data with Artemis. Brief Bioinform.

[B43] TMHMM Server v. 2.0: Prediction of transmembrane helices in proteins. http://www.cbs.dtu.dk/services/TMHMM/.

[B44] Emanuelsson O, Brunak S, von Heijne G, Nielsen H (2007). Locating proteins in the cell using TargetP, SignalP, and related tools. Nature Protocols.

[B45] Mulder N, Apweiler R (2007). InterPro and InterProScan: tools for protein sequence classification and comparison. Methods Mol Biol.

[B46] Carver TJ, Rutherford KM, Berriman M, Rajandream MA, Barrell BG, Parkhill J (2005). ACT: the Artemis Comparison Tool. Bioinformatics.

